# Supplementary figures and images for: NIEND: neuronal image enhancement through noise disentanglement
Source: Bioinformatics. 2024 Mar 26;40(4):btae158. doi: 10.1093/bioinformatics/btae158 (PMC11650625; doi:10.1093/bioinformatics/btae158)

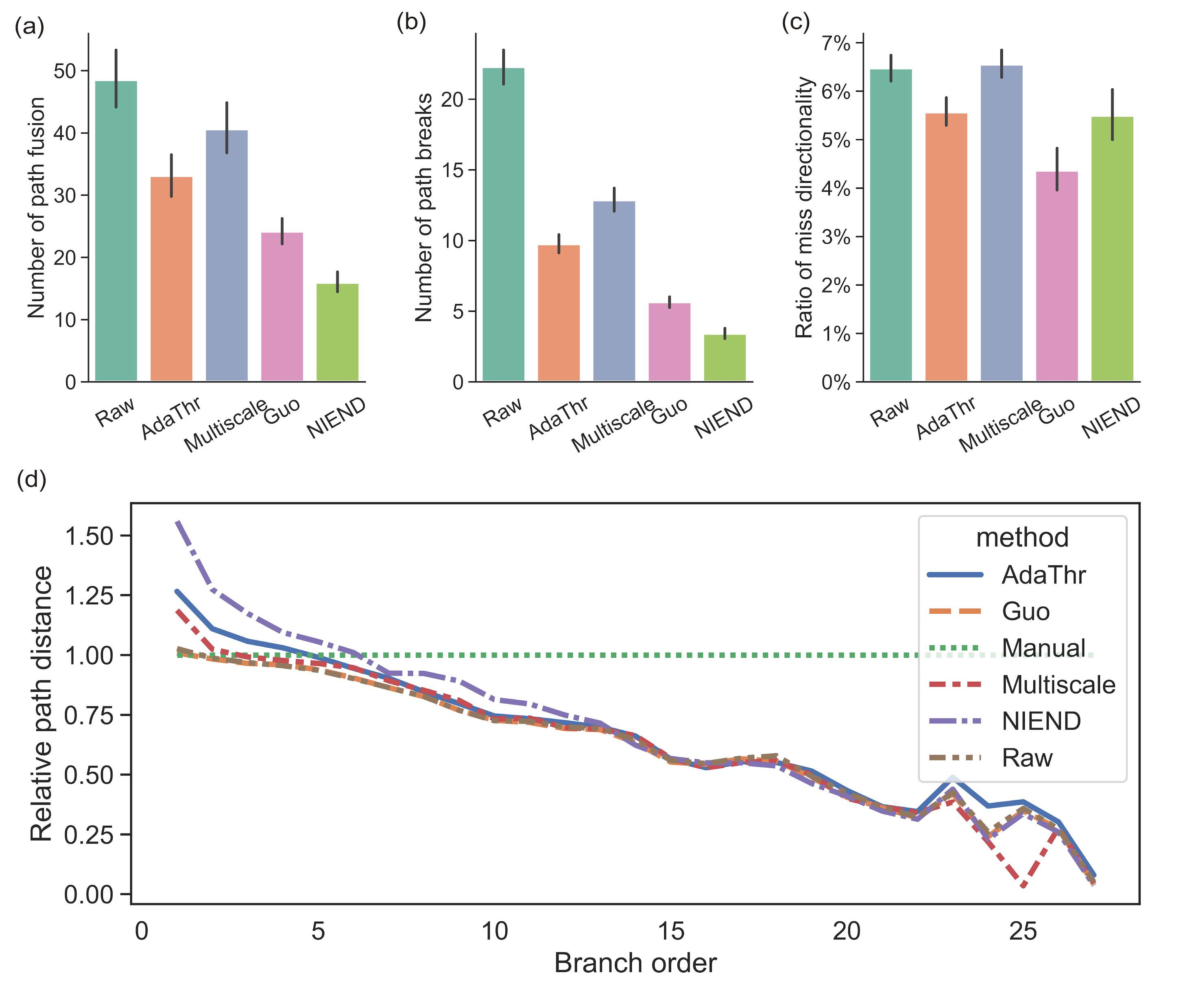

Supplement: btae158_Supplementary_Data [file btae158_supplementary_data.zip › S6.png]

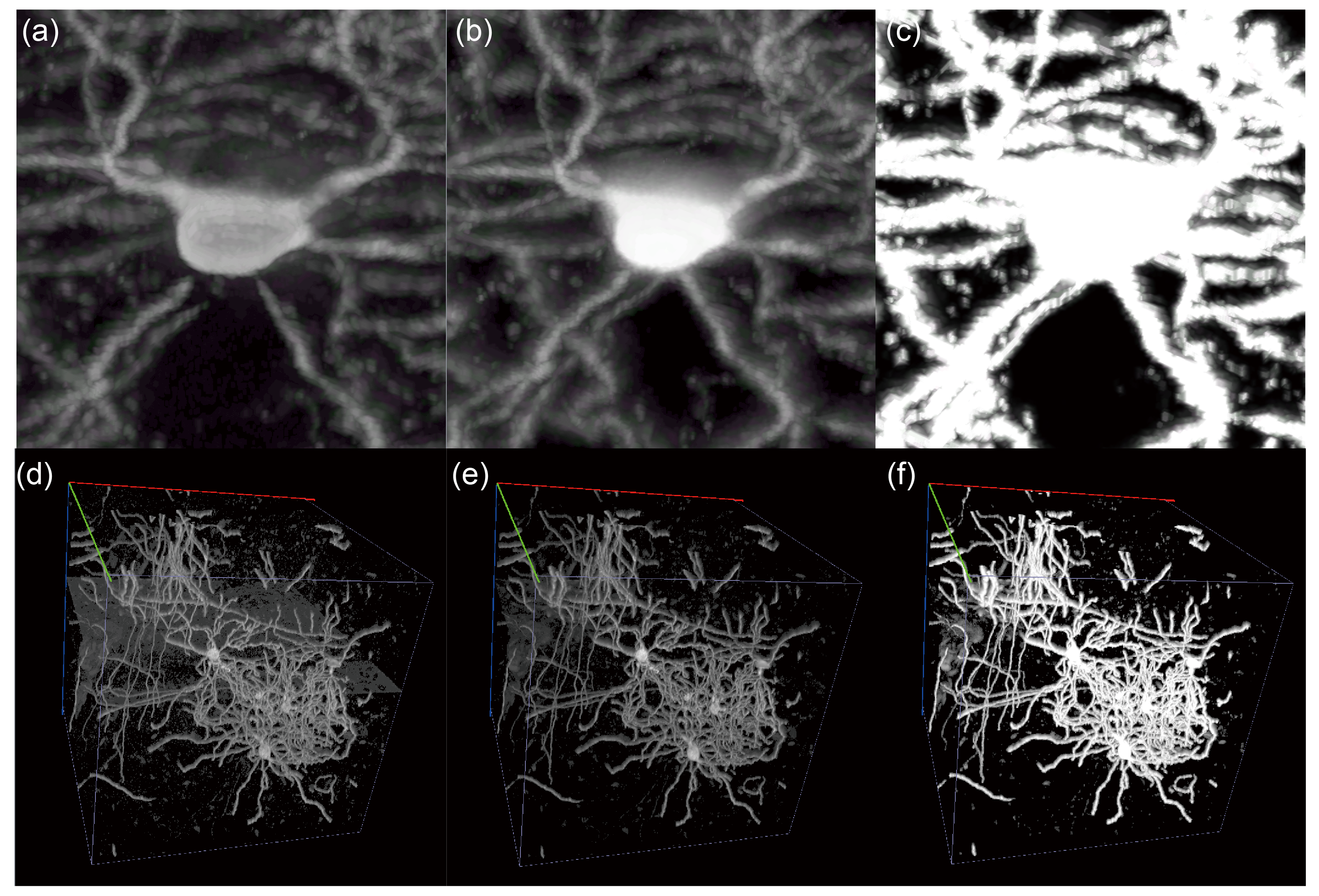

Supplement: btae158_Supplementary_Data [file btae158_supplementary_data.zip › S3.png]

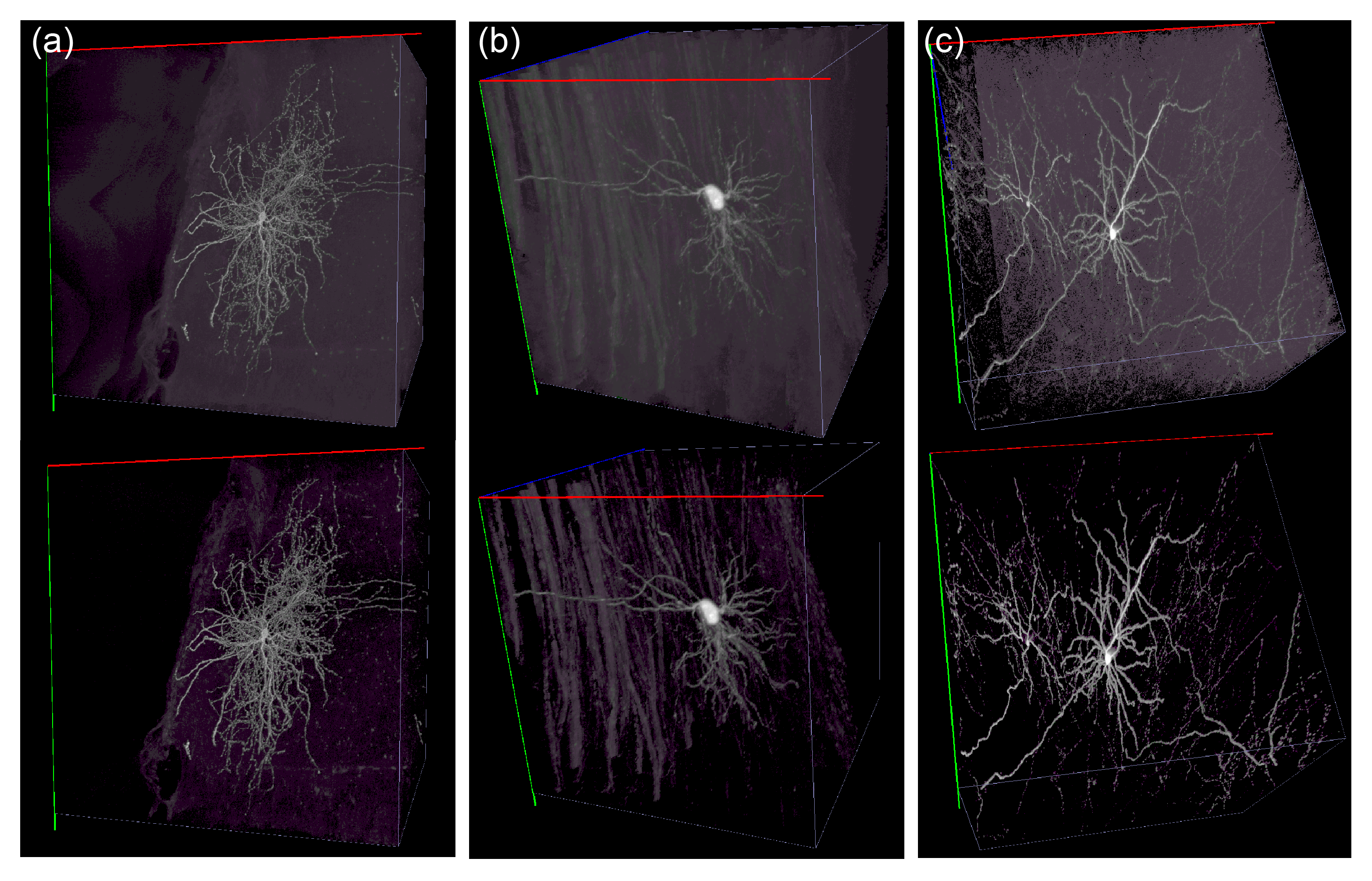

Supplement: btae158_Supplementary_Data [file btae158_supplementary_data.zip › S4.png]

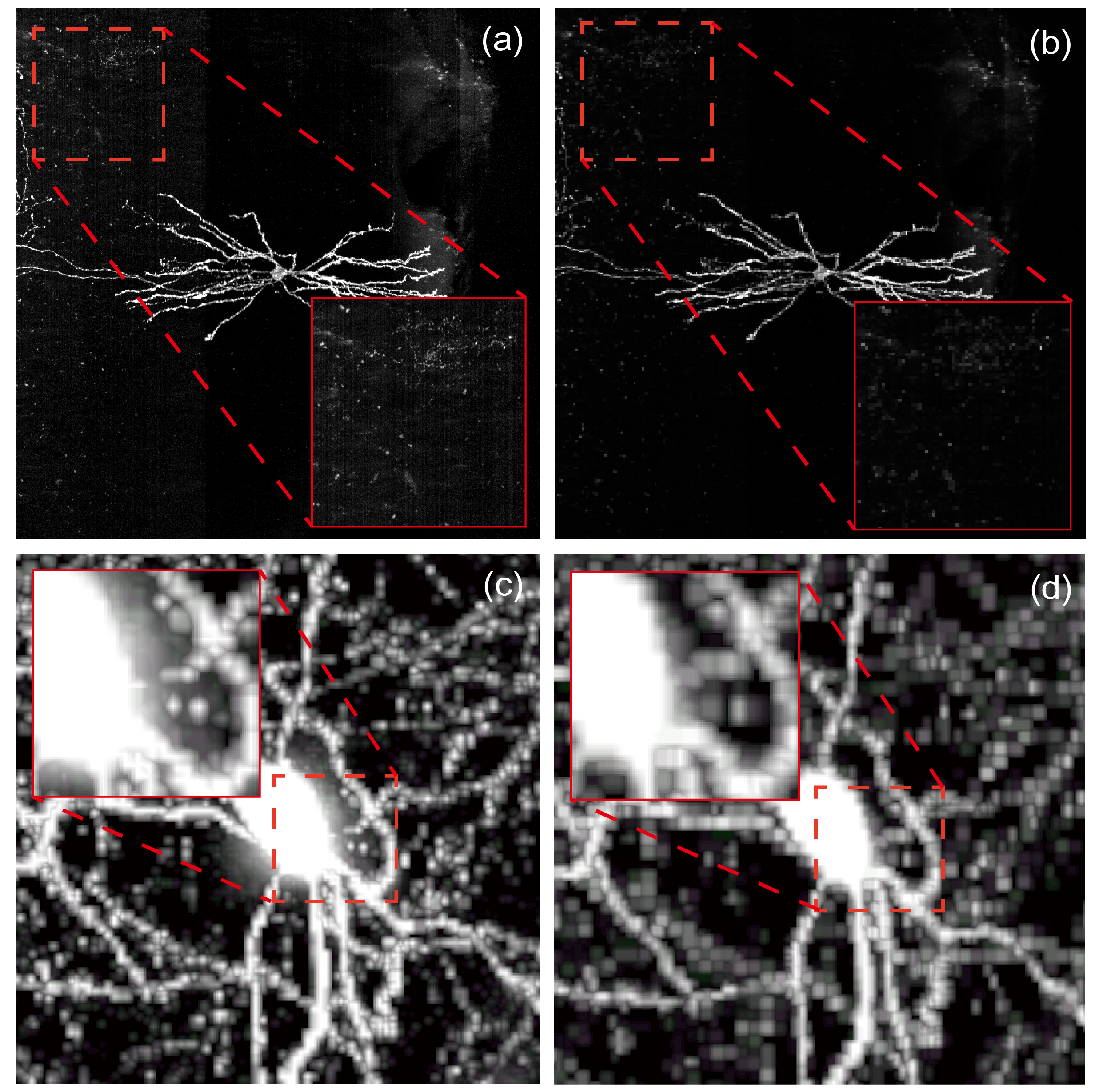

Supplement: btae158_Supplementary_Data [file btae158_supplementary_data.zip › S2.png]

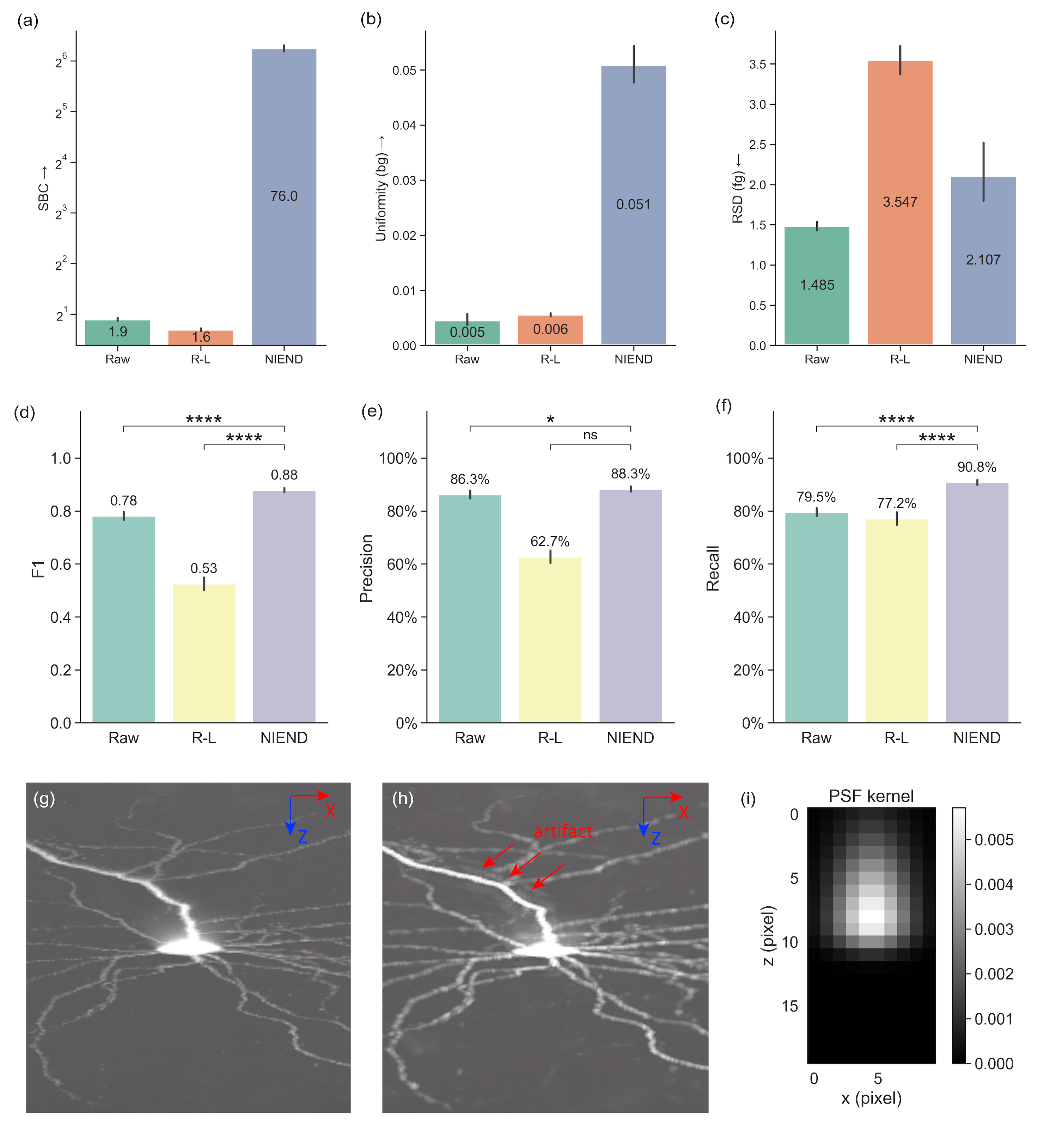

Supplement: btae158_Supplementary_Data [file btae158_supplementary_data.zip › S9.png]

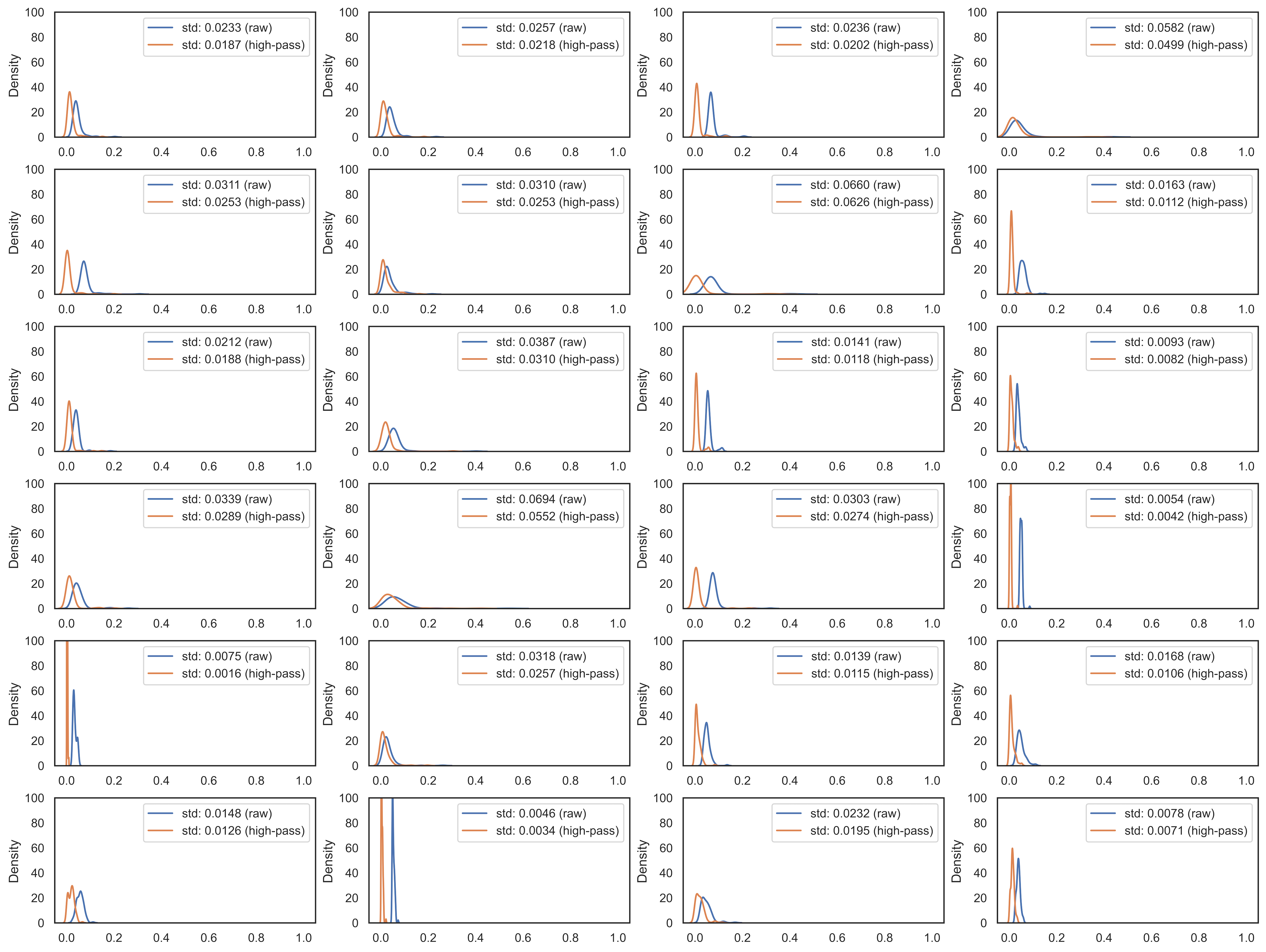

Supplement: btae158_Supplementary_Data [file btae158_supplementary_data.zip › S5.png]

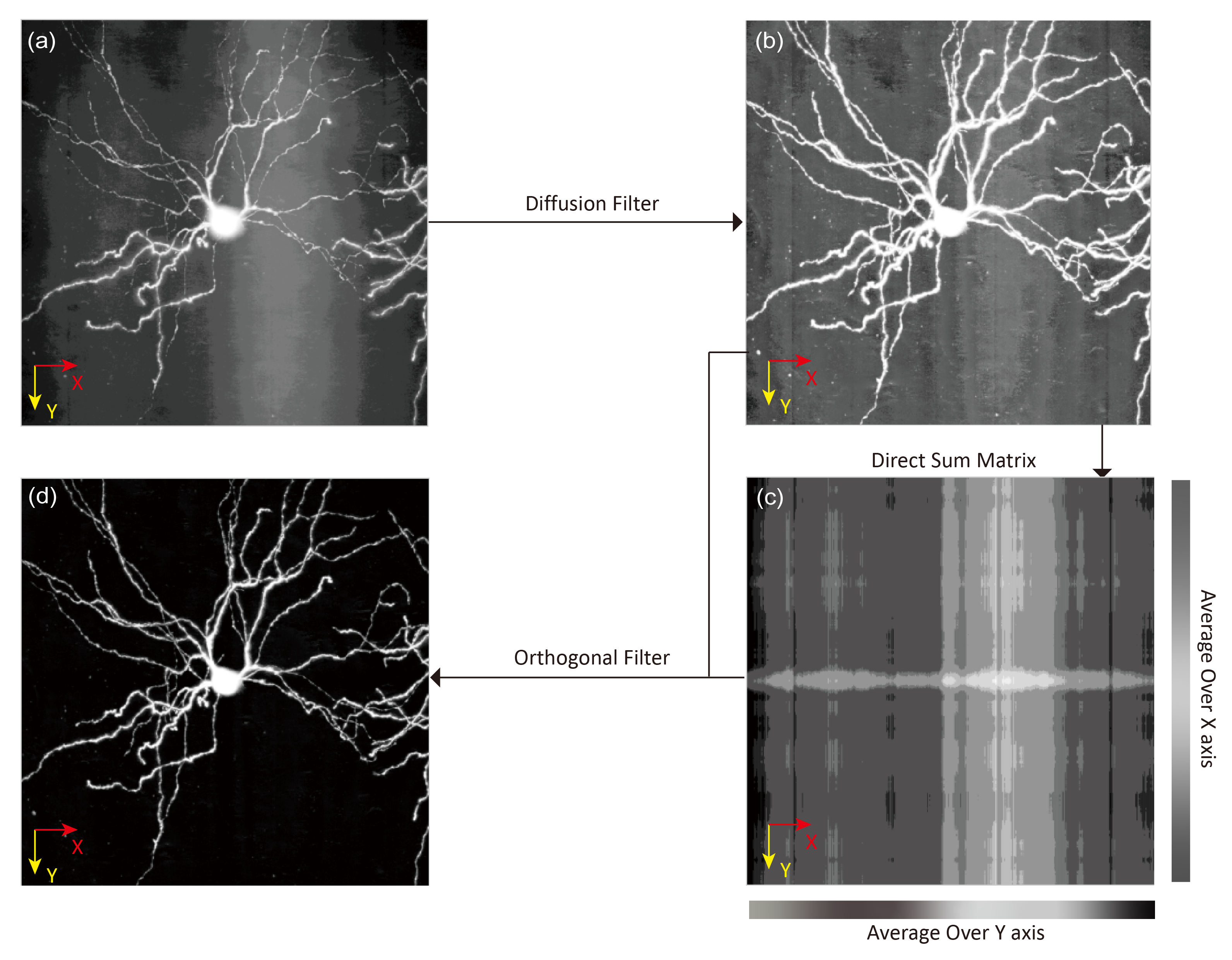

Supplement: btae158_Supplementary_Data [file btae158_supplementary_data.zip › S1.png]

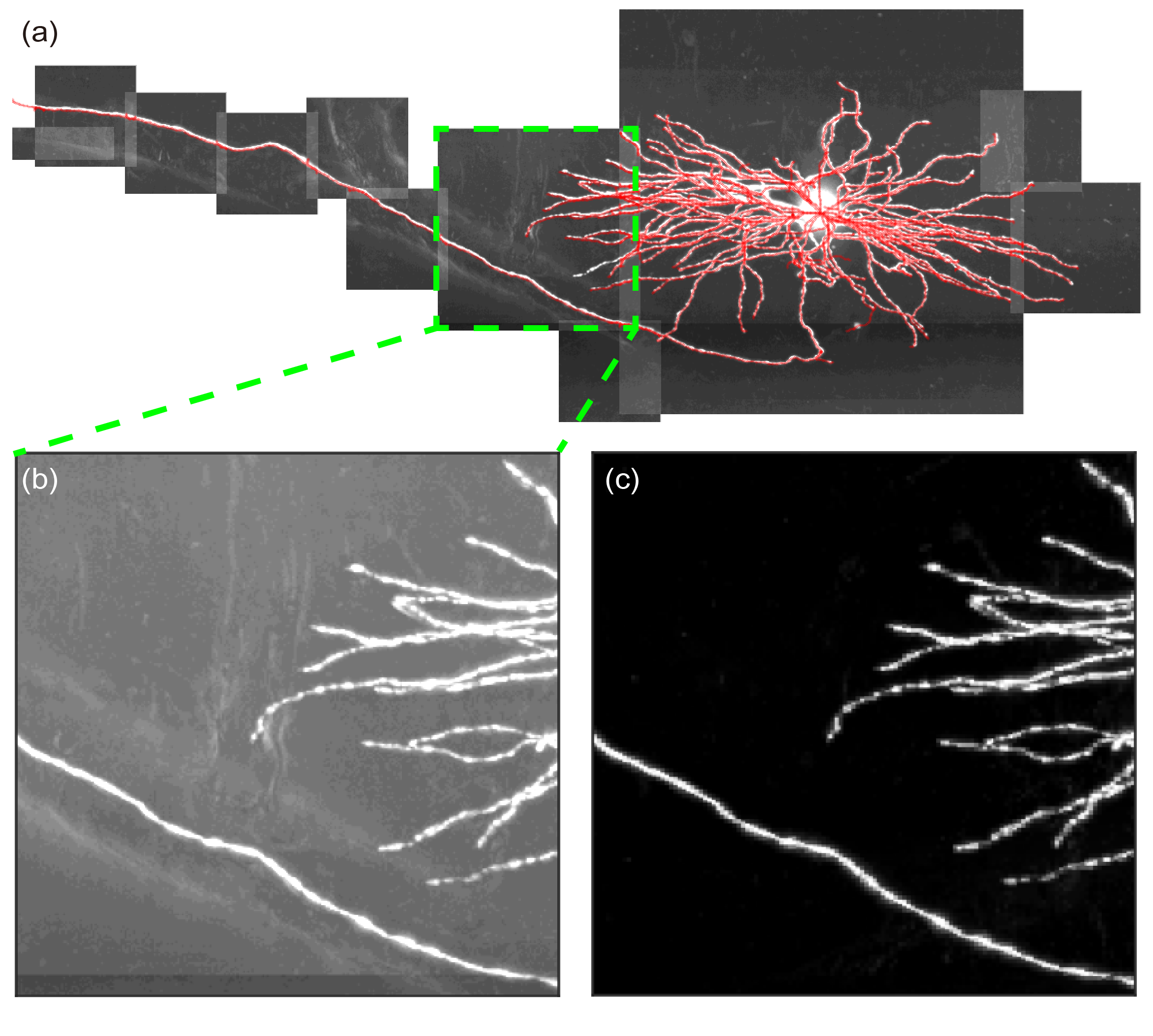

Supplement: btae158_Supplementary_Data [file btae158_supplementary_data.zip › S7.png]

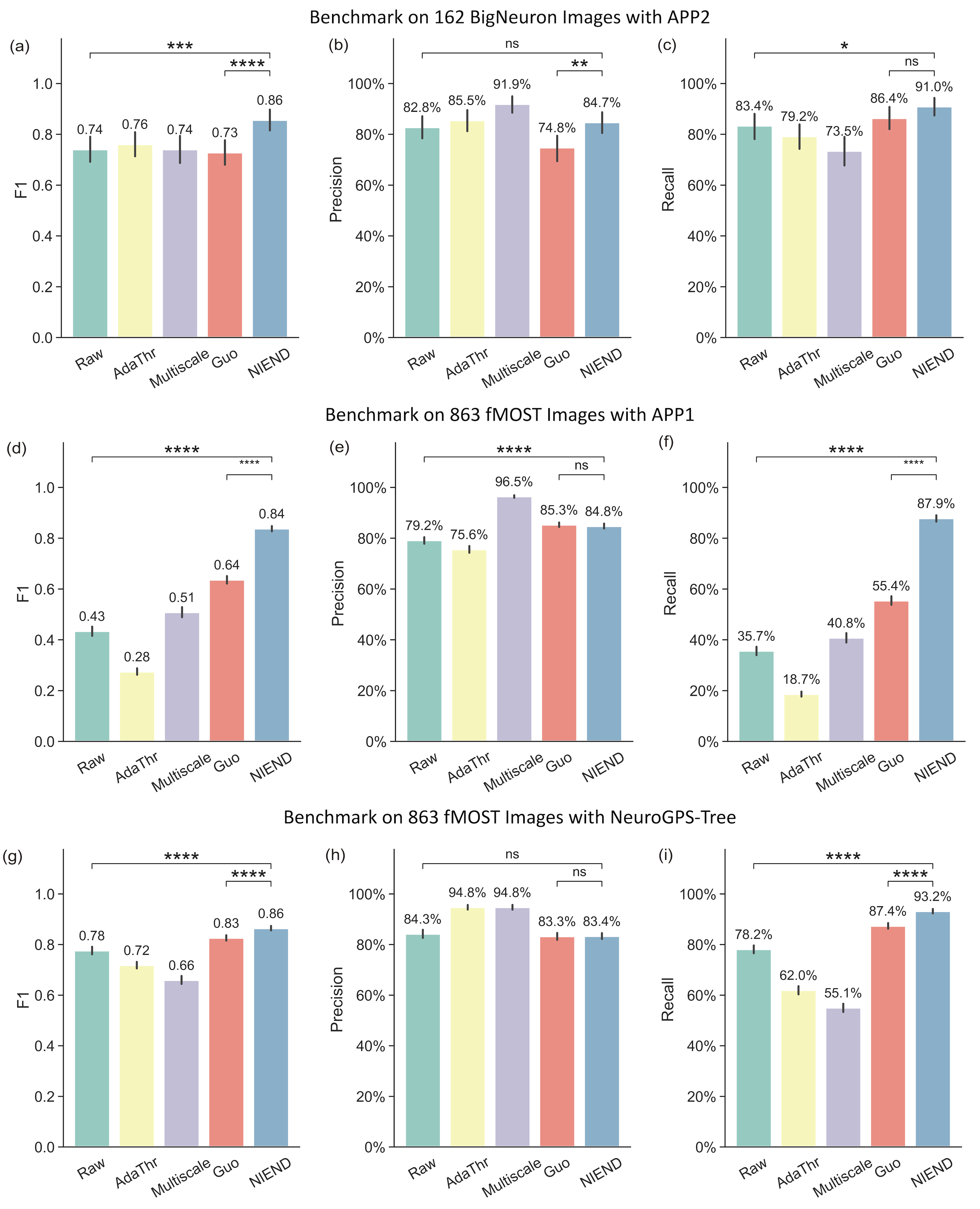

Supplement: btae158_Supplementary_Data [file btae158_supplementary_data.zip › S8.png]
